# An Herbal Drug, *Gongjin-dan*, Ameliorates Acute Fatigue Caused by Short-Term Sleep-Deprivation: A Randomized, Double-Blinded, Placebo-Controlled, Crossover Clinical Trial

**DOI:** 10.3389/fphar.2018.00479

**Published:** 2018-05-11

**Authors:** Mi Ju Son, Hwi-Jin Im, Boncho Ku, Jun-Hwan Lee, So Young Jung, Young-Eun Kim, Sung Bae Lee, Jun Young Kim, Chang-Gue Son

**Affiliations:** ^1^Clinical Research Division, Korea Institute of Oriental Medicine, Daejeon, South Korea; ^2^Liver and Immunology Research Center, Daejeon Korean Medicine Hospital of Daejeon University, Daejeon, South Korea; ^3^KM Fundamental Research Division, Korea Institute of Oriental Medicine, Daejeon, South Korea; ^4^Korean Medicine Life Science, University of Science & Technology, Daejeon, South Korea; ^5^Mibyeong Research Center, Korea Institute of Oriental Medicine, Daejeon, South Korea

**Keywords:** herbal medicine, *Gongjin-dan*, fatigue, sleep, randomized controlled trial

## Abstract

**Introduction:** *Gongjin-dan* (GJD) is an herbal drug commonly used in Korea and China to combat fatigue, but there are only few clinical studies on its effectiveness and experimental studies on its mechanism of action, and no randomized controlled trial of GJD on the efficacy and mechanism of action has been reported. Here, we performed an exploratory study to evaluate both questions regarding GJD use in humans.

**Methods:** A randomized, double-blinded, placebo-controlled, crossover clinical trial was conducted in the Republic of Korea. Healthy male participants were recruited and randomly allocated to groups receiving GJD-placebo or placebo-GJD in sequence. Fatigue was artificially induced by sleep deprivation for 2 nights. The primary outcome was a change in serum cortisol level; levels of biomarkers for stress hormones as well as oxidative stress and immunologic factors were also assessed, and questionnaires on fatigue and sleep quality were conducted.

**Results:** Twelve and 11 participants were assigned to the GJD-placebo and placebo-GJD groups, respectively. Of all 23 participants, depending on crossover design, we analyzed a total of 20 participants for GJD, and 21 for placebo. An increase in serum cortisol appeared to be attenuated by GJD administration (*p* = 0.25), but the effect was not statistically significant; a similar pattern was observed in salivary cortisol levels (*p* = 0.14). Overall, GJD showed a tendency to reduce fatigue according to the Brief Fatigue Inventory (BFI, *p* = 0.07) and the Fatigue Severity Scale (FSS, *p* = 0.13) questionnaires. BFI and FSS scores in the first stage (before the crossover), however, were significantly improved (BFI, *p* = 0.02; FSS, *p* = 0.05) after GJD treatment (relative to placebo). GJD also seemed to improve sleep quality as assessed by the Leeds Sleep Evaluation Questionnaire (*p* = 0.06), with a significant improvement specifically in the condition “Getting To Sleep” (*p* = 0.02). Five participants experienced minor adverse events, but no adverse events were specific to the GJD administration period.

**Conclusions:** This trial produced the first clinical evidence that GJD might have anti-fatigue properties, especially under sleep deprivation; however, the investigation of cortisol-mediated mechanisms requires further larger-scale studies in the future.

Trial registration: World Health Organization International Clinical Trials Registry Platform KCT0001681 (http://apps.who.int/trialsearch/Trial2.aspx?TrialID=KCT0001681).

## Introduction

Fatigue is a perceived sense of tiredness or lack of energy that is sufficient to negatively affect daily life; one large survey indicated a prevalence of fatigue of about 20% in the general population (Hannay, 1978; Kant et al., 2003). Prolonged fatigue reduces quality of life in areas such as occupational performance and cognitive function (De Meirleir and McGregor, 2003). Lost productivity associated with chronic fatigue has been estimated to cost between £75 and £129 million annually in the United Kingdom (Collin et al., 2011), and about $136 million annually in United States (Ricci et al., 2007).

Some studies suggest that sleep is closely associated with fatigue. Acute sleep deprivation can impair mood, cognitive performance, and psychomotor function (Philibert, 2005; Ikegami et al., 2009), and a prolonged lack of sleep can reduce work productivity and lead to occupational burnout (Baldwin and Daugherty, 2004; Ekstedt et al., 2006). However, the causal relationship between fatigue and sleep has not yet been elucidated.

Recently, an interaction between sleep deprivation and fatigue with regard to circadian rhythms has been hypothesized. The known circadian rhythm of cortisol levels relates closely to daily physiological functions such as sleep propensity, alertness, and cognitive performance (Lac and Chamoux, 2003; Wright et al., 2015). Circadian rhythmicity and sleep, on the other hand, play important roles in the regulation of plasma cortisol concentrations via regulation of the hypothalamo-pituitary-adrenal axis (Guyon et al., 2014; Wright et al., 2015).

*Gongjin-dan* (GJD) is an herbal medicine broadly used throughout Korea and China to combat fatigue-related disorders (Lee et al., 2013). GJD is considered to be effective in reversing a lack of energy, as well as in the prevention of disease, according to the classical medical text *DongUiBoGam* (Heo, 2013); it has been approved by the Ministry of Food and Drug Safety (MFDS) of the Republic of Korea, where it is commercially marketed for poor health, fatigue, and fatigue-related disorders. In animal studies, GJD demonstrated anti-fatigue, antioxidant, anti-inflammatory, and neuroprotective properties (Choi and Park, 2007; Hur et al., 2008; Sunwoo et al., 2012; Hong et al., 2015). Despite the many clinical experiences supporting the use of GJD and the evidence from animal studies, there is no randomized controlled trials evaluating the anti-fatigue effects of GJD and its underlying mechanism.

We hypothesized that GJD may improve fatigue symptoms by regulating cortisol and other stress hormones, as well as by regulating oxidative stress and immunological activity. This study aimed to investigate the anti-fatigue effect of GJD, by applying short-term sleep deprivation to healthy volunteers and analyzing biomarkers of cortisol activity and other stress hormones as well as markers for oxidative and immunological activities, in a controlled crossover experiment.

## Materials and methods

This study was designed and conducted based on the principles of the Declaration of Helsinki, with the patient's rights and well-being in mind. The study protocol, which had been published previously (Son et al., 2016), was approved by the institutional review board of the Daejeon Korean Medicine Hospital of Daejeon University (DJOMC-127-1), and was registered with the national clinical trial registry “Clinical Research Information Service,” which is the primary registry of the World Health Organization International Clinical Trials Registry Platform in the Republic of Korea (http://apps.who.int/trialsearch/Trial2.aspx?TrialID=KCT0001681; CRIS registration No. KCT0001681).

### Study design and participants

This was a randomized, double-blinded (both patient and practitioner or assessor), placebo-controlled crossover clinical trial conducted in the Republic of Korea. Healthy male participants aged 19–45 years were recruited using online and printed advertisements. Written informed consent was obtained from all participants.

The primary inclusion criteria were: normal sleep pattern and time, non-smoker or ex-smoker who stopped smoking at least 1 year before the study; the primary exclusion criteria were: a clinically significant medical history, laboratory test results showing alanine aminotransferase or aspartate aminotransferase levels of more than twice the upper limit of the normal, and alcohol or drug use, which could affect the results. A full list of inclusion and exclusion criteria is provided in the protocol (Son et al., 2016).

Participants were instructed not to eat foods that might affect cortisol levels, such as those high in sugar, fat, or protein, for 12 h before the first administration of investigational drugs (Gibson et al., 1999; MacKenzie et al., 2007). During the study period, participants were not allowed to receive other fatigue-related treatment such as dietary supplements or similar alternatives, and were required to consume the same foods. To minimize possible bias, all participants were limited to intensive exercise.

### Randomization and blinding

Randomization codes were generated through block randomization using Random Allocation Software v2.0.0 (M. Saghaei, MD, Department of Anesthesia, Isfahan University of Medical Sciences, Isfahan, Iran). A researcher who was not involved in recruitment or assessment assigned randomization codes to the subjects. The number was printed on a piece of paper, put in an opaque, sealed envelope, and stored in a double-locked cabinet. The randomization code was attached after the manufacturing and packaging of GJD and placebo. A researcher not involved in the previous steps opened the envelopes sequentially and assigned an individual participant to each identification number. The opened envelopes were also stored separately in a double-locked cabinet. Participants, practitioners, and outcome assessors were blinded to the treatment.

### Procedures

Eligible participants who voluntarily signed the informed consent form were recruited. The participants were instructed to sleep at least 7 h per night for 1 week before admission and to avoid new medications, alcohol, and caffeine consumption for 2 days before admission. Participants were randomly allocated to one of two groups; group allocation was concealed from both the participants and the practitioners.

For both sequential groups, participants were hospitalized for 4 days, and after a washout period of 4 weeks, the participants were hospitalized again for 4 days. During hospitalization, subjects were administered GJD or placebo for 3 days. The participants took GJD or placebo 1 pill twice a day 30 min before breakfast and dinner, a total of six times during hospitalization. A practitioner checked that participants took the GJD/placebo immediately after administration, and collected the empty bottles, in order to confirm that all doses had been taken.

We have defined the two time periods of hospitalization as Phase I (initial intake) and Phase II (second intake). In both phases, participants were allowed to sleep for only 4 h (02:00–06:00) on days 2 and 3 to artificially induce fatigue; daytime sleep was limited by engaging the participants in various activities such as walking, taking lectures, and watching movies. Blood samples were collected at 06:30 for analysis of serum cortisol, stress hormones, oxidative stress-related biomarkers, homocysteine, and immunological factors. Saliva samples were collected at 06:30, 12:00, and 21:00 for analysis of salivary cortisol, while heart rate variability (HRV) was assessed between 15:00 and 17:00. Self-reporting of the Korean versions of the Fatigue Severity Scale (FSS), the Brief Fatigue Inventory (BFI), the Leeds Sleep Evaluation Questionnaire (LSEQ), and a Daily Sleep and Fatigue Status questionnaire was carried out via a questionnaire distributed after dinner on day 1, 2, 3, and 4.

After Phase I, participants underwent a 4-week wash-out period, and were then admitted to Phase II for 4 days. One group received GJD in Phase I, then placebo in Phase II; the other group received placebo first, then GJD.

### Interventions

The trial medications were prepared by Kyoung-Bang Pharmaceutical Co. Ltd (Incheon, Republic of Korea), certified from the MFDS in good manufacturing practices of herbal medicine extract pills and granules. Quality control (Supplementary Figure 1) and assurance of the quality and safety testing, the packaging, and the contents of the treatment interventions including checks for potential contaminants such as heavy metals or steroids, were undertaken by the manufacturers to ensure treatment drug stability and quality.

GJD consists of three medicinal herbs (*Ginseng radix, Korean angelica, Corni fructus*), two animal-derived materials *(Cornus cervi parvum, Moschus)* and *Mel*. The raw herbs were washed, dried, ground, mixed with a pure form of honey (Mel), and formed into pills. All constituents of this formulation complied with Korean Pharmacopoeia standards.

A placebo was manufactured by the same pharmaceutical company, consisting of corn starch as the main ingredient, as well as small amounts of *Dioscoreae Rhizoma, Poria Sclerotium, Mel*, a coloring agent, and a flavoring agent. The GJD and the placebo pills wrapped in gold foil were formulated to a similar size, shape, taste, and flavor, and all weighed 3.75 g each.

The chemical components of GJD have been described in our previous article (Hong et al., 2015). Ginsenoside Rg1 and Rb1 from *G. radix*, morronoside and loganin from *Cornus cerviparvum*, nodakenin and decrusin from *Korean Angelica*, and L-muscone from the *Moschus* were detected in GJD by fingerprinting analysis.

### Outcomes

The primary outcome was a change in serum cortisol levels. Because of its natural circadian rhythm, serum cortisol was measured on an empty stomach 30 min after waking (at 06:30).

Salivary cortisol levels were measured once at admission on day 1 at 21:00, and three times daily on day 2, 3, and 4 (within 30 min of waking, at 12:00, and at 21:00). A blood sample for analysis of epinephrine, norepinephrine, levels of oxidative stress-related biomarkers [reactive oxygen species (ROS), nitric oxide (NO), malondialdehyde (MDA), protein carbonyl, glutathione (GSH), GSH reductase (GSH-Rx), superoxide dismutase (SOD), catalase, and total antioxidant capacity (TAC)], homocysteine, and levels of immunological factors [tumor necrosis factor (TNF)-α, interferon (IFN)-γ, interleukin (IL)-2, IL-10, IL-12, T-cells, B-cells, and natural killer (NK) cells] was collected at the same time as the serum cortisol. The previously validated self-report questionnaires described above (see section Procedures) were utilized to measure perceived fatigue. The FSS was used to measure overall status of fatigue in the past week (Krupp et al., 1989), the BFI for variation in daily fatigue (Mendoza et al., 1999; Yun et al., 2005), and the LSEQ for variations in sleep characteristics (Kim et al., 2014). The LSEQ involves four domains related to sleep: Getting To Sleep (GTS), Quality Of Sleep (QOS), Awakening From Sleep (AFS), and Behavior Following Wakefulness (BFW).

To complement the results of the BFI and the LSEQ, a Daily Sleep and Fatigue Status questionnaire developed by our research team was also completed by the participants once a day from day 2 to day 4.

Every afternoon on day 1–day 4, HRV testing was performed to study autonomic nervous system responses relevant to fatigue. For HRV assessments, 5 min of electrocardiogram signal were measured and analyzed using a Neo DINAMIKA system (MR Co. Ltd., Gyeonggi-do, Republic of Korea).

### Laboratory analysis

Serum and salivary levels of cortisol, as well as serum levels of epinephrine and norepinephrine, were assessed using enzyme-linked immunosorbent assay (ELISA) kits (LDN GmbH & Co., Nordhorn, Germany).

Total serum levels of ROS were determined according to Hayashi's method (Hayashi et al., 2007), and serum NO levels were determined using the Griess method (Green et al., 1982). MDA was determined using thiobarbituric acid-reactive substances (Kamal et al., 1989), and the serum levels of protein carbonyl were measured using a commercial ELISA kit (Cell Biolabs Inc., San Diego, CA). The serum levels of total GSH and GSH-Rx were determined using commercial kits (Abcam, Cambridge, MA), and SOD activity in the serum was determined using a SOD assay kit (Dojindo Laboratories, Kumamoto, Japan) according to the manufacturer's protocol. Serum catalase activity and TAC were assayed as previously described (Wheeler et al., 1990; Kambayashi et al., 2009). Serum homocysteine levels were analyzed using an auto-analyzer (AU400; Olympus, Tokyo, Japan).

Serum levels of immune-related cytokines were measured using commercial ELISA kits for TNF-α, IFN-γ, as well as IL-2, IL-10, and IL-12 (BD Biosciences, San Jose, CA). All absorbance measurements were carried out using a spectrophotometer (Molecular Devices, Sunnyvale, CA).

Lymphocyte subclasses in the peripheral blood were analyzed using a BD Multitest IMK kit with a FACSCalibur instrument and Cell Quest Pro software (BD Biosciences). Complete blood counts were obtained using a HEMAVET analyzer (CDC Technologies, Dayton, OH). T cells (CD3+), B cells (CD19+), and NK cells (CD3−/CD16+/CD56+) were quantified as a percentage of the total number of lymphocytes.

### Sample size and statistical analysis

No previous clinical trials for serum cortisol levels from which sample size could be calculated have been reported; however, 24 was considered a practical number of participants from which to detect clinically important differences between treatment and control groups. This number of subjects (12 per group) is the recommended minimum number per group for pilot studies (Julious, 2005).

An independent statistician who was blinded to the randomized allocation of participants carried out the statistical analysis. Measured variables including primary and secondary outcomes were assessed using the full analysis set (FAS) based on intention-to-treat (ITT) principles. Normality was tested by using the Shapiro–Wilk test.

The baseline characteristics were summarized using means and standard deviations for the continuous variables if satisfying normality, or using median and interquartile range for non-normal data. The baseline difference between both sequential groups was assessed using an independent two-sample *t*-test or Wilcoxon's rank sum test. For all measured values/scores, the temporal difference (i.e., the difference in the value measured between the endpoint and baseline values) was calculated and statistically analyzed; the mean temporal differences were also analyzed between the GJD and the placebo group.

For all crossover end points, the analysis was performed using a linear mixed effects model adjusted for baseline, sequence, and study period covariates, and a carry-over effect was calculated using the Grizzle model (Grizzle, 1965). The results are shown as least squares means and 95% confidence intervals (CI). The level of significance was set to 0.05 (two-tailed), and all analyses were performed with the software package R (version 3.2.4, “R & R” of the Statistics Department of the University of Auckland, Auckland, New Zealand).

## Results

### Study participants

**Figure 1:**
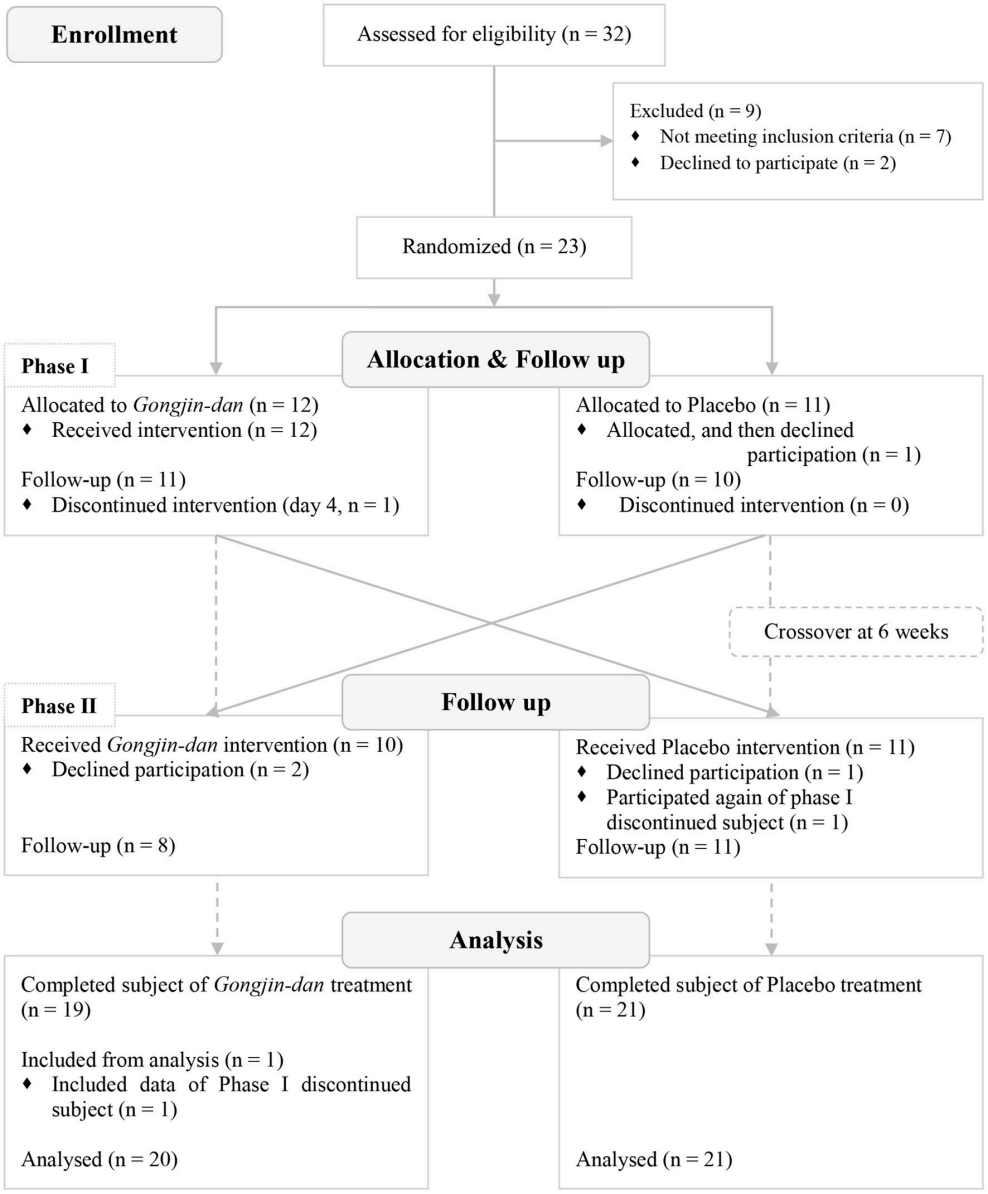
CONSORT flow diagram of subjects.

A total of 32 participants were screened; 23 subjects were randomized to this trial. Of these, 12 subjects were placed in the GJD-placebo sequential group, and 11 were placed in the placebo-GJD sequential group. One participant withdrew from the placebo-GJD sequential group before the first treatment due to unknown reasons. One participant from the GJD-placebo sequential group did not complete the treatment on day 4 of Phase I, due to personal reasons not relevant to the trial, but participated again in Phase II. Thus, 21 subjects completed the trial. According to FAS based on ITT analysis, a total of 20 participants receiving GJD treatment, and 21 participants receiving placebo treatment were successfully analyzed (Figure 1). At baseline, there were no statistically significant differences between the sequential groups regarding any variable (Table 1). Carry-over effects were not found.

**Table 1:** Demographic characteristics of the study participants.

	**Group A (*N* = 12)**	**Group B (*N* = 10)**	***p*-value**
Age (year)	22.8 ± 2.5	23.9 ± 2.8	0.36
Height (cm)	173.0 ± 4.4	172.3 ± 6.2	0.76
Weight (kg)	73.0 ± 10.4	68.8 ± 6.4	0.28
BMI	24.3 ± 2.6	23.2 ± 2.4	0.30
**Blood Pressure (mmHg)**			
Systolic	124.9 ± 7.0	124.0 ± 10.4	0.81
Diastolic	66.5 [59.5–77.0]	71.5 [65.0–80.0]	0.22
Pulse (beats/min)	70.5 [65.5–75.0]	76.0 [66.0–81.0]	0.12
Body Temperature (°C)	36.6 ± 0.3	36.8 ± 0.4	0.18
FSS	3.0 ± 1.1	2.6 ± 0.8	0.31
BFI	2.2 [1.8–4.3]	2.1 [1.8–4.4]	1.00
PSQI	5.0 [3.0–5.0]	3.0 [3.0–4.0]	0.17

*Group A: participants were administered Gongjin-dan in Phase I, followed by placebo treatment in Phase II; Group B: participants were administered placebo in Phase I, followed by Gongjin-dan treatment in Phase II*.

*Participants data that met the full analysis set were used for demographic analysis*.

*Independent two-sample t-tests or Wilcoxon's rank sum test were used for statistical analysis and results are reported as mean ± standard deviation (SD) or median [lower interquartile range–upper interquartile range]*.

*BMI, Body mass index; FSS, Fatigue Severity Scale; BFI, Brief Fatigue Inventory; PSQI, Pittsburgh Sleep Quality Index*.

### Serum and salivary cortisol

**Figure 2:**
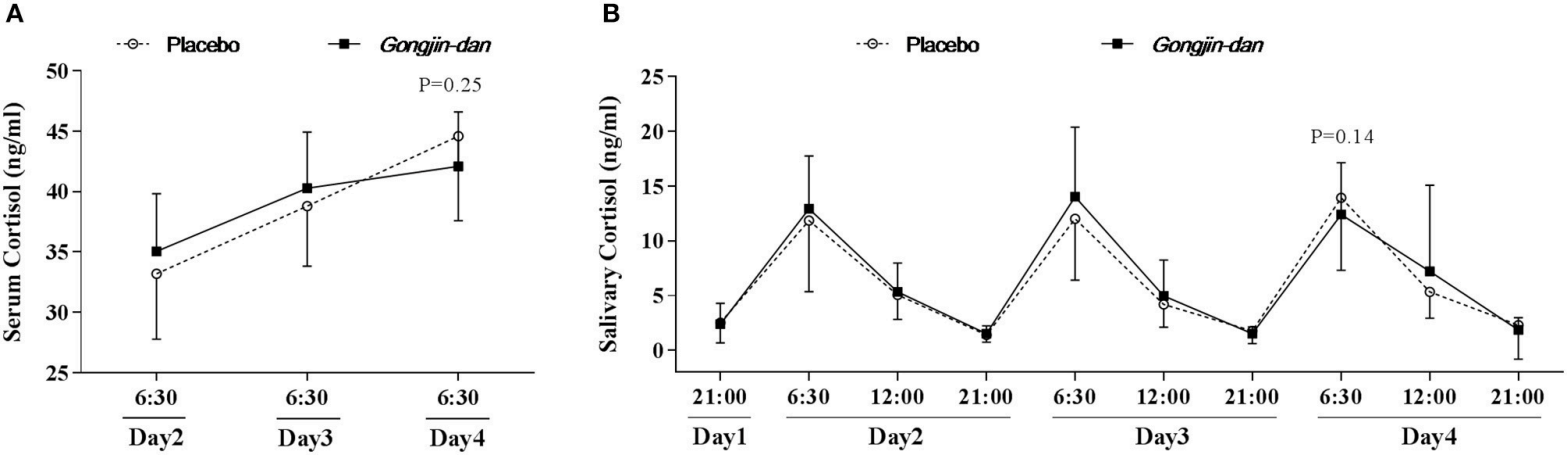
Daily variations in serum & salivary cortisol. Results are expressed as means with 95% confidence intervals (CIs). *P*-values indicate significance of mean differences in the value (from endpoint to baseline) between treatment groups. Linear mixed effect models were used for statistical analysis. **(A)** Serum cortisol level. **(B)** Salivary cortisol level.

There was a small, non-significant difference in serum cortisol levels, in that the magnitude of the increase in serum cortisol appeared to be attenuated by GJD administration. GJD treatment caused an increase of 7.2 ng/ml [95% CI: 2.16–12.25], and the placebo treatment caused an increase of 11.23 ng/ml [95% CI: 6.53–15.93], as tested by a linear mixed-effect model (*p* = 0.25; Figure 2A). The elevation in salivary cortisol evaluated at 06:30 every day was similar to that seen for the serum cortisol (GJD: −3.14 [95% CI: 2.01–1.27], Placebo: −0.32 [95% CI: 4.5–1.19], *p* = 0.14; Figure 2B).

### Changes in fatigue scores

**Figure 3:**
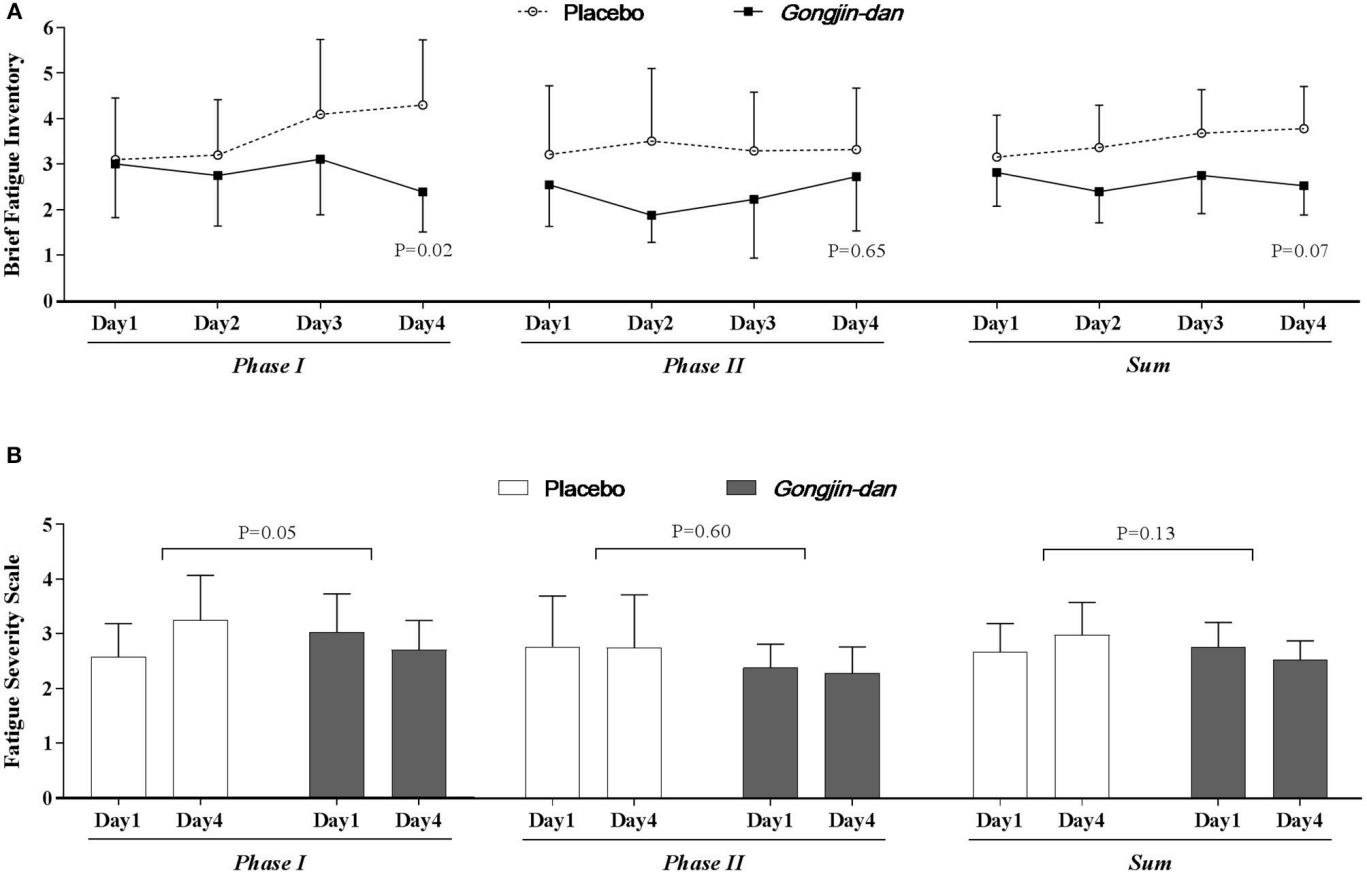
Anti-fatigue effect of *Gongjin-dan*. Results are means with 95% CIs. *P*-values indicate the significance of mean differences in the temporal change in value (endpoint-baseline) between treatment groups. Linear mixed-effect models were used for statistical analysis. **(A)** Brief fatigue inventory. **(B)** Fatigue severity scale.

BFI scores improved with GJD treatment relative to placebo, but statistical significance was borderline (GJD difference: −0.16 [95% CI: −0.90 to 0.59], placebo difference: 0.83 [95% CI: 0.13–1.53], *p* = 0.07; Figure 3A). The change in FSS from baseline was −0.1 [95% CI: −0.54 to 0.33] and 0.36 [95% CI: −0.05 to 0.77] for the GJD and placebo treatment, respectively (*p* = 0.13; Figure 3B).

The BFI score significantly decreased with GJD treatment, whereas it increased with placebo treatment in Phase I (GJD: −0.32 [95% CI: −1.19 to 0.54], placebo: 1.28 [95% CI: 0.37–2.18], *p* = 0.02). The FSS score also showed a significant difference in Phase I (GJD: −0.09 [95% CI: −0.59 to 0.41], placebo: 0.62 [95% CI: 0.10–1.15], *p* = 0.05). The BFI and FSS scores in Phase II showed no significant differences, either between groups or from baseline to endpoint values. The Daily Fatigue Status questionnaire produced results similar to the BFI.

### Changes in sleep characteristics

**Figure 4:**
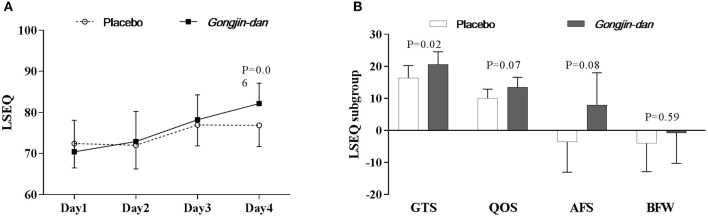
Sleep quality effect of *Gongjin-dan*. **(A)** LSEQ score, results are means with 95% CIs. **(B)** LSEQ subgroup, results are the least squares means of the temporal differences (endpoint – baseline) with 95% CIs. Linear mixed-effect models were used for statistical analysis. Least squares means from linear mixed-effects models were adjusted for baseline, sequence, and study period covariates. AFS, Awakening From Sleep; BFW, Behavior Following Wakefulness; GTS, Getting To Sleep; LSEQ, Leeds Sleep Evaluation Questionnaire; QOS, Quality Of Sleep.

LSEQ scores increased, but only with borderline significance, in the GJD treatment group relative to placebo (GJD difference: 10.3 [95% CI: 5.47–15.12], placebo difference: 5.07 [95% CI: 0.51–9.63], *p* = 0.06; Figure 4A).

However, when analyzed by LSEQ subgroup, GTS scores significantly improved with GJD treatment vs. placebo (*p* = 0.02). GJD treatment showed a tendency to improve QOS and AFS scores, but significance was borderline (QOS, *p* = 0.07; AFS, *p* = 0.08; Figure 4B). The Daily Sleep Status questionnaire produced results similar to the LSEQ.

### Changes in stress hormones and oxidative stress biomarkers

ROS values were decreased more significantly from baseline to endpoint with GJD treatment relative to placebo (GJD changed value: −8.76 [95% CI: −14.5 to −3.03], placebo changed value: −1 [95% CI: −6.46 to 4.47], *p* = 0.01). However, no other tested biomarkers showed significant differences between GJD and placebo treatment (Table 2).

**Table 2:** Stress hormones and oxidative stress biomarkers.

**Outcomes**	***Gongjin-dan***		**Placebo**		**Treatment difference**
	**Endpoint^†^**	**Change from baseline^†^^‡^**	**Endpoint^†^**	**Change from baseline^†^^‡^**	
**STRESS HORMONE**					
Epinephrine (pg/ml)	24.2 (17.9, 30.6)	−9.2 (−15.5, −2.9)	26.1 (20.1, 32.0)	−7.4 (−13.3, −1.4)	−1.9 (−10.7, 7.0)
Norepinephrine (pg/ml)	476.5 (368.1, 585.0)	16.9 (−91.5, 125.4)	488.0 (388.9, 587.1)	28.4 (−70.7, 127.5)	−11.5 (−165.1, 142.1)
**OXIDATIVE STRESS BIOMARKER**					
ROS (unit/ml)	112.7 (107.0, 118.4)	−8.8 (−14.5, −3.0)	120.5 (115.0, 126.0)	−1.0 (−6.5, 4.5)	−7.8^*^ (−13.8, −1.8)
NO (μM/l)	8.6 (6.5, 10.6)	3.5 (1.4, 5.6)	10.1 (8.2, 12.1)	5.1 (3.1, 7.0)	−1.6 (−4.3, 1.1)
MDA (μM/l)	10.2 (6.8, 13.6)	−0.3 (−3.7, 3.1)	9.1 (5.9, 12.3)	−1.4 (−4.5, 1.9)	1.1 (−3.3, 5.4)
Protein carbonyl (ηM/mg protein)	64.0 (57.1, 71.0)	8.0 (1.1, 14.8)	59.4 (53.0, 65.9)	3.3 (−3.1, 9.8)	4.6 (−4.3, 13.5)
GSH (μM/ml)	8.9 (5.8, 12.0)	1.8 (−1.2, 4.9)	7.4 (4.6, 10.3)	0.4 (−2.5, 3.2)	1.5 (−2.6, 5.6)
GSH Reductase (μM/ml)	18.4 (16.7, 20.0)	−0.2 (−1.8, 1.4)	19.6 (18.0, 21.1)	1.0 (−0.5, 2.6)	−1.2 (−3.0, 0.6)
SOD (unit/ml)	2.5 (2.2, 2.9)	−0.3 (−0.6, 0.1)	2.7 (2.4, 3.0)	−0.1 (−0.4, 0.2)	−0.2 (−0.7, 0.3)
Catalase (unit/ml)	296.3 (251.9, 340.6)	59.2 (14.9, 103.5)	330.4 (289.0, 371.9)	93.4 (52.0, 134.8)	−34.2 (−93.9, 25.5)
TAC (unit/ml)	393.3 (359.6, 427.0)	46.0 (12.3, 79.7)	395.4 (363.7, 427.1)	48.2 (16.4, 79.9)	−2.2 (−42.8, 38.5)
Homocysteine (μmol/l)	15.3 (14.2, 16.3)	−0.3 (−1.4, 0.7)	16.1 (15.2, 17.1)	0.5 (−0.4, 1.5)	−0.9 (−2.4, 0.6)

*Results are reported as least squares means with 95% CIs. Least squares means from linear mixed-effects models are adjusted for baseline, sequence, and study period covariates*.

† *All outcomes were measured at 06:30 on day 2 (baseline) and day 4 (endpoint), respectively*.

‡ *Change from baseline is the value at endpoint minus the value at baseline*.

* *p = 0.01*.

GSH, glutathione; MDA, malondialdehyde; NO, nitric oxide; ROS, reactive oxygen species; SOD, superoxide dismutase; TAC, total antioxidant capacity

### Other secondary outcomes

There were no significant differences in HRV or measured immunological factors (TNF-α, IFN-γ, IL-2, IL-10, IL-12, T-cells, B-cells, and NK cells levels) between GJD and placebo treatment.

### Adverse events

Of 22 subjects, five subjects experienced adverse events. Two subjects complained of headache; one experienced the headache during both GJD and placebo treatment, while the other subject experienced headache only during placebo treatment. Two subjects complained of abdominal distension; one experienced it in both treatment conditions, while the other subject experienced it only during placebo treatment. One subject experienced constipation in both GJD and placebo treatment periods. One subject had high systolic blood pressure (from 140 to 150 mmHg) during both study periods. These symptoms all fully resolved within 2 days of the end of the experimental treatment period without any event-specific intervention. No adverse events specific to the GJD treatment periods only were reported, and no serious adverse events were reported.

## Discussion

The hypothesis tested in this study was that GJD would exert anti-fatigue effects by regulating levels of cortisol, in accordance with the results seen in a previous animal experiment (Hong et al., 2015). GJD failed to significantly inhibit the fatigue-induced increase in cortisol seen in the placebo group. However, the GJD treatment group appeared to show a small attenuation in its cortisol increase (*p* = 0.25). One reason for the relative lack of effect may be the timing of the treatment. We assumed that the onset of treatment effects in terms of cortisol levels was prior to or by the beginning of day 3, whereas the endpoint value was calculated at 06:30 on day 4. As seen in Figure 2A, the slope of the line representing cortisol during GJD treatment flattens off at day 3, relative to placebo treatment. This result suggests that a peak effect was not achieved. In addition, the total of four pills of GJD treatment, chosen because the endpoint value of serum cortisol was evaluated before drug administration on day 4, may not be adequate to reach a peak effect. Further studies evaluating the onset, peak, and duration of effects of GJD are needed to determine the mechanism underlying GJD, not to mention to determine accurate prescription guidelines. Lastly, without prior studies to draw from, we could have selected an inappropriate sample size, which would reduce our statistical power.

GJD tended to improve perceived fatigue (BFI and FSS scores), although with borderline significance. The effect was especially significant in Phase I, but not in Phase II. It is possible that fatigue might not be induced by the sleep deprivation in Phase II, because participants could have adapted to the study schedule; this would be consistent with our findings.

GJD gradually improved sleep quality during the study period. Among the LSEQ subgroups, subjects taking GJD reported getting to sleep to be significantly easier than those taking placebo; subjects taking GJD also tended to have improved quality of sleep and fewer times awakening from sleep relative to those taking placebo. These results indicate that GJD may ameliorate acute fatigue and improve sleep quality related to acute sleep deprivation.

The molecular mechanisms underlying fatigue are not well understood due to the complicated nature of its causation. However, recent studies have suggested the involvement of oxidative stress, as well as involvement of the endocrine, metabolic, autonomic nervous, and immune systems, in causing fatigue (Jason et al., 2009; Maes and Twisk, 2010). It has been reported that in fatigued individuals relative to healthy (non-fatigued) individuals, circulating oxidative stressors are increased, while antioxidant levels are decreased (Vecchiet et al., 2003; Kennedy et al., 2005; Kim et al., 2010). In particular, ROS levels were correlated to the severity of fatigue, and were shown to be very high in subjects diagnosed with chronic fatigue syndrome (Kennedy et al., 2005). In our subjects, serum ROS levels were significantly reduced with GJD treatment (*p* = 0.01), similar to results of a previous study (Hong et al., 2015). These findings indicate that suppressing oxidative stress with anti-oxidant drugs or supplements might be effective in alleviating fatigue. The anti-oxidant effects of GJD have been described in other animal studies (Choi and Park, 2007; Hong et al., 2015). Our results suggest that anti-oxidant activity in GJD may suppress fatigue by regulating serum ROS levels in human blood.

Serious adverse events did not occur in this study, indicating that GJD is safe for short-term use (up to 3 days at least). Interestingly, the minor adverse events that occurred were not specific to the GJD treatment, but were instead also found during placebo treatment. Thus, these may not be related to the GJD treatment itself, but they may instead be related to fatigue induced by sleep deprivation or individual reactions to the change of the participant's living environment.

This was a novel study in that it used a pre-designed model in which subjects were hospitalized and fatigued in an artificial way. We believe that this design is applicable to clinical research, and that 4 h of daily sleep for 2 days is sufficiently little time for inducing fatigue even if the sleep deprivation is not administered repeatedly. The attempt to evaluate fatigue and drug effects on fatigue with objective outcome measures, in addition to subjective ones (e.g., questionnaires) is also a relatively novel idea.

There are several limitations to this study. First, the small sample size is likely a weakness of this study, given the number of our statistical results that were borderline. This represents, however, a feasibility (pilot) study investigating the anti-fatigue effect of GJD; therefore, no previous clinical trials were available to perform a statistical power assessment and adjust the sample size accordingly. We judged that a pilot study recommendation was applicable to this study (Julious, 2005), but further studies with larger sample sizes are needed to draw definitive conclusions. Based on this result, a sufficient sample size for assessing serum cortisol levels would be 61 per group, whereas 23 and 35 participants in each group would be needed for evaluating BFI and FSS, respectively.

Second, while the placebo was the same size and shape as the GJD pill, and all participants were told beforehand that they would be receiving GJD, there was a possibility of a loss of participant blinding because the flavor of the placebo did indeed differ slightly from that of GJD. The results of a blinding questionnaire showed that 15 of 19 participants (78.9%) chose the correct test sample; nine of 15 in the correct group, and one of four in the incorrect group replied “Its [the sample's] flavor and taste seem more like a real drug.” Six of the 15 correct respondents, and three of the four incorrect respondents, replied “It is more effective.” The unique formulations of traditional herbal drugs (not to mention the strong flavors of many plant alkaloids and other products) make it difficult to produce adequate placebos. Future work is needed to develop a unique placebo that can also mimic the flavor of the treatment drug when traditional herbal medicines are being tested.

Third, because fatigue was artificially induced in this study, the findings are difficult to transfer to conditions such as chronic fatigue syndrome and idiopathic chronic fatigue, which are more clinically relevant than artificial sleep deprivation. Moreover, we included only male participants, to ensure high internal validity and to reduce possible confounding factors. It might therefore be questionable to generalize the results to the female population. To evaluate the effects of GJD in the real world, future studies will need to recruit patients regardless of their sex. Furthermore, studies with a reasonably large sample size of patients with chronic fatigue and appropriate outcome evaluations such as fatigue questionnaires and anti-oxidative biomarkers are needed in the future.

## Conclusions

In conclusion, this trial tentatively supports the empirical clinical evidence of the anti-fatigue ability of GJD, especially under conditions of sleep deprivation. This study is the first clinical trial of GJD as an anti-fatigue agent, and represents a unique opportunity to enhance our understanding of fatigue and the effects of GJD on fatigue with regard to endocrinological and immunological mechanisms.

## Ethics statement

This study was carried out in accordance with the rules, regulations, and guidelines of Ministry of Food and Drug Safety of Republic of Korea with written informed consent from all subjects. All subjects gave written informed consent in accordance with the Declaration of Helsinki. The protocol was approved by the institutional review board of the Daejeon Korean Medicine Hospital of Daejeon University. This study protocol was published in Trials (Trials 2016 17:418, https://doi.org/10.1186/s13063-016-1542-7).

## Author contributions

C-GS: Contributed to the conception and design of the study; MS: Wrote the manuscript; H-JI: Participated in the design of the study and helped to draft the manuscript; BK: Conducted statistical design and analysis of the study; MS, H-JI, Y-EK, SL, and JK: Performed the experiments; SJ: Monitored data collection; J-HL: Provided technical advice and contributed to a critical review of the manuscript. All authors read and approved the final manuscript.

### Conflict of interest statement

The authors declare that the research was conducted in the absence of any commercial or financial relationships that could be construed as a potential conflict of interest.
