# Supplementary material for: An Herbal Drug, Gongjin-dan, Ameliorates Acute Fatigue Caused by Short-Term Sleep-Deprivation: A Randomized, Double-Blinded, Placebo-Controlled, Crossover Clinical Trial
Source: Front Pharmacol. 2018 May 11;9:479. doi: 10.3389/fphar.2018.00479 (PMC5958722; doi:10.3389/fphar.2018.00479)
Supplement: Supplementary file 1 [file Presentation_1.PPTX]

## Slide 1
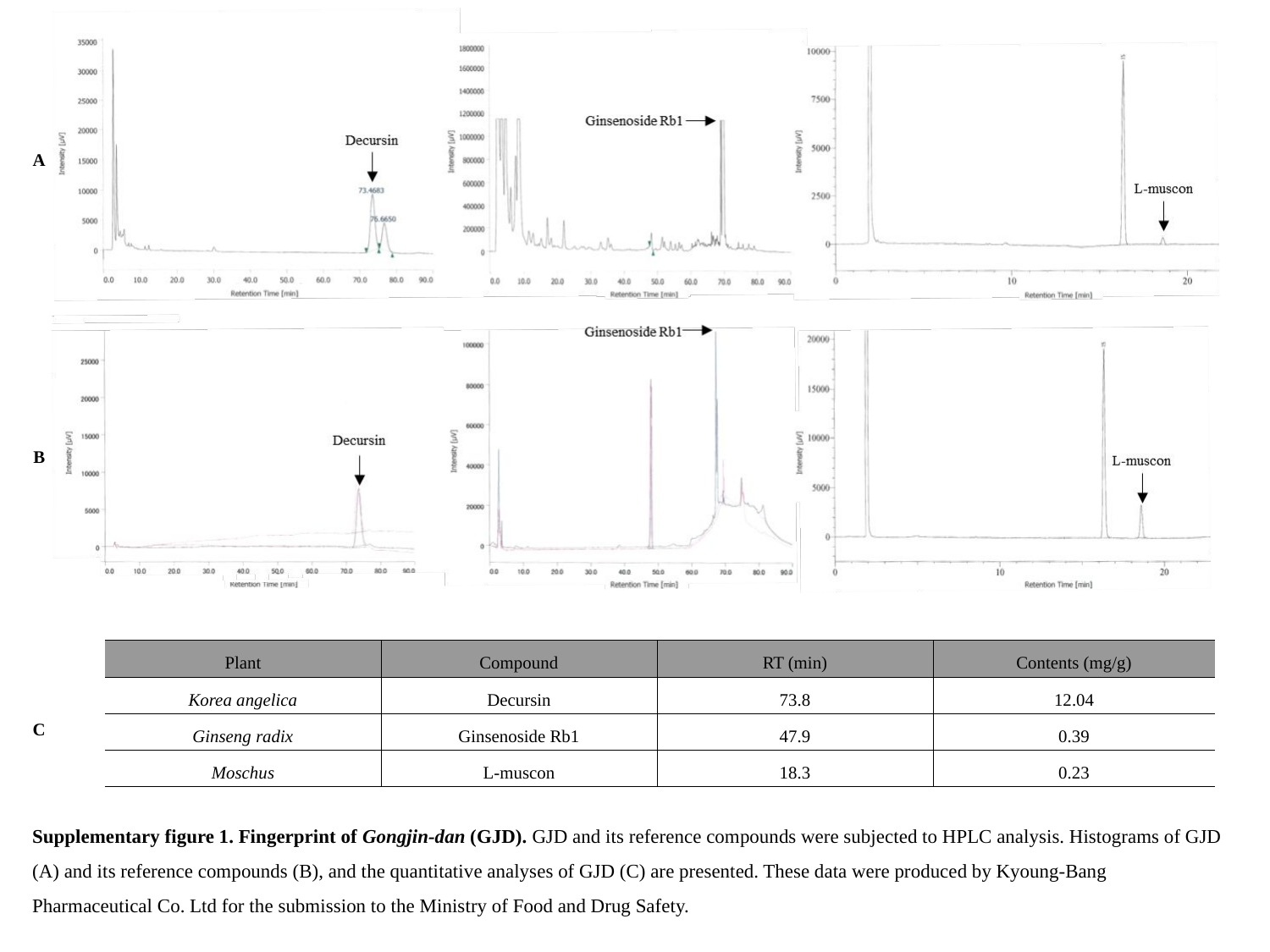

A
B
| Plant | Compound | RT (min) | Contents (mg/g) |
| --- | --- | --- | --- |
| Korea angelica | Decursin | 73.8 | 12.04 |
| Ginseng radix | Ginsenoside Rb1 | 47.9 | 0.39 |
| Moschus | L-muscon | 18.3 | 0.23 |
C
Supplementary figure 1. Fingerprint of Gongjin-dan (GJD). GJD and its reference compounds were subjected to HPLC analysis. Histograms of GJD (A) and its reference compounds (B), and the quantitative analyses of GJD (C) are presented. These data were produced by Kyoung-Bang Pharmaceutical Co. Ltd for the submission to the Ministry of Food and Drug Safety.
